# Altered Pancreatic Islet Function and Morphology in Mice Lacking the Beta-Cell Surface Protein Neuroligin-2

**DOI:** 10.1371/journal.pone.0065711

**Published:** 2013-06-11

**Authors:** Charles Zhang, Arthur T. Suckow, Steven D. Chessler

**Affiliations:** 1 Department of Medicine, University of California Irvine, School of Medicine, Irvine, California, United States of America; 2 Pediatric Diabetes Research Center, University of California San Diego, La Jolla, California, United States of America; University of Bremen, Germany

## Abstract

Neuroligin-2 is a transmembrane, cell-surface protein originally identified as an inhibitory synapse-associated protein in the central nervous system. Neuroligin-2 is also present on the pancreatic beta-cell surface, and there it engages in transcellular interactions that drive functional maturation of the insulin secretory machinery; these are necessary for normal insulin secretion. The effects of neuroligin-2 deficiency on brain and neuronal function and morphology and on behavior and coordination have been extensively characterized using neuroligin-2 knockout mice. The effects of absent neuroligin-2 expression on islet development and function, however, are unknown. Here, to help test whether neuroligin-2 is necessary for normal islet development, we characterized islet morphology in mice lacking neuroligin-2. To test whether–as predicted by our earlier co-culture studies–absence of neuroligin-2 impairs beta cell function, we compared glucose-stimulated insulin secretion by islets from mutant and wild-type mice. Our results show that while islets from neuroligin-2-deficient mice do not to appear to differ architecturally from wild-type islets, they are smaller, fewer in number, and contain beta cells with lower insulin content. Evaluation of transcript levels suggests that upregulation of neuroligin-1 helps compensate for loss of neuroligin-2. Surprisingly, under both basal and stimulating glucose levels, isolated islets from the knockout mice secreted more of their intracellular insulin content. Rat islets with shRNA-mediated neuroligin-2 knockdown also exhibited increased insulin secretion. Neurexin transcript levels were lower in the knockout mice and, consistent with our prior finding that neurexin is a key constituent of the insulin granule docking machinery, insulin granule docking was reduced. These results indicate that neuroligin-2 is not necessary for the formation of pancreatic islets but that neuroligin-2 influences islet size and number. Neuroligin-2–perhaps through its effects on the expression and/or activity of its binding partner neurexin–promotes insulin granule docking, a known constraint on insulin secretion.

## Introduction

Normal pancreatic beta cell function depends on the proximity of beta cells to other beta cells: on beta-cell to beta-cell contact [1], [2]. In part this is because gap junctions at sites of contact are necessary for the coordination of insulin secretion [2], [3]. Proximity may also be important by virtue of facilitating beta-cell-to-beta-cell paracrine signaling [2]. It is becoming increasingly clear that clustering of beta cells is also necessary in order for transcellular interactions integral to the maturation and normal functioning of the insulin secretory machinery to take place between proteins on the beta-cell surface. Two transcellular protein interactions that directly influence functioning of the insulin secretory machinery have been described: EphA-ephrin-A interactions and neuroligin-2 interactions [4], [5]. The insulin secretory and synaptic neurotransmitter machineries bear close resemblance to each other, so it is perhaps not surprising that EphA/ephrin-A and neuroligin are both neuronal proteins that, through interactions across the synaptic cleft, are important for synaptic maturation and secretory function [6], [7], [8]. The binding of EphA on one beta cell with ephrin-A on a neighboring beta cell contributes to the suppressed basal insulin secretion and enhanced glucose-stimulated secretion observed when dissociated beta cells are brought back into contact with each other [4]. While transcellular E-cadherin interactions also affect insulin secretion, it is not known whether this is due to the protein’s importance for cell adhesion and/or an effect on gap junction formation or rather, as with EphA/ephrin and neuroligin-2, due to a more direct interaction with the insulin secretory machinery [9], [10]. The work described here focuses on the synaptic adhesion protein neuroligin-2.

Neuroligin-2 was first identified as a central nervous system protein localized to the postsynaptic side of inhibitory synapses [11], [12]. Through protein-protein interactions across the synaptic cleft, neuroligins help spur synaptic functional maturation and are afterwards necessary for the ongoing maintenance of normal synaptic function [7], [11] We previously demonstrated that neuroligin-2 is expressed in pancreatic islets in a beta-cell specific manner and that it influences insulin secretion [13]. More recently we found that, like ephrin-A/EphA, neuroligin-2 engages in transcellular (beta-cell-to-beta-cell) interactions that are necessary for the normal functioning of the insulin secretory machinery [5]. Analogous to its function in inhibitory synapses, neuroligin-2’s role is, at least in part, to drive maturation of the beta-cell subplasmalemmal secretory machinery. These studies were carried out primarily in a co-culture model in which beta cells were brought into contact with HEK293 cells expressing neuroligin-2 [5].

The neuronal and behavioral phenotypes of neuroligin-2 knockout mice have been extensively characterized [11], [14], [15], [16], [17]. After we found that neuroligin-2 is expressed in beta cells and identified its importance for insulin secretion in our co-culture system, it was natural to next probe the effects of neuroligin-2 deficiency on islet formation and function in the knockout mice.

In order better understand the role of neuroligin-2 in beta-cell function, we analyzed insulin secretion by islets from the mutant mice. Our previous findings in the co-culture model had led us to predict that neuroligin-2 deficiency would cause impairment of insulin secretion. Our earlier studies also provided a hint that neuroligin-2 may be important for islet formation: in addition to its effects on assembly of the insulin secretory machinery, neuroligin-2 increased INS-1 beta-cell proliferation [5]. The knockout mice afforded the opportunity to determine, in vivo, the effects on islet formation of the absence of neuroligin-2 throughout development. Since mutations in neuroligin are associated with some cases of autism and schizophrenia, knowledge of the phenotypic effects of neuroligin-2 deletion may have direct clinical relevance [18], [19], [20], [21], [22].

Here we report that neuroligin-2 knockout results in decreased islet number, islet size, and beta cell insulin content. In addition to providing evidence for a role of neuroligin-2 in islet formation, these findings complement previous studies of the neuroligin-2 knockout mouse, demonstrating that neuroligin-2 deficiency has phenotypic effects on a tissue outside the central nervous system. In islets from the knockout mice, neuroligin-2 deficiency resulted in increased neuroligin-1 expression and decreased neurexin expression. Consistent with decreased beta-cell neurexin expression, insulin granule docking was reduced, and insulin secretion was increased. Prolonged, shRNA-mediated neuroligin-2 knockdown in cultured islets also increased insulin secretion. These results contribute further evidence that neuroligin-2 modulates beta cell function. Our findings suggest a model in which, in contrast to the acute effects observed previously in the co-culture system, over the longer term, compensatory mechanisms–perhaps including increased neuroligin-1 expression and/or reduced neurexin expression and granule docking–counter the impairment in insulin secretory capacity caused by neuroligin-2 deficiency.

## Materials and Methods

### Islet Isolation and Culture

Neuroligin-2 knockout mice were kindly provided by Frederique Varoqueaux (Max Planck Institute for Experimental Medicine). The generation of these mice has previously been described [11]. Experiments were performed using neuroligin-2 null (Nlgn2^−/−^) mice and wild-type (WT) littermate controls generated by the breeding of heterozygous pairs. Islets were isolated from WT and neuroligin-2 knockout (KO) mice and also from Sprague-Dawley rats (240–260 g; Charles River Laboratories) using collagenase (Liberase, Roche) as previously described [13], [23], [24]. Islets were allowed to recover for 24 h in culture after isolation. Culture medium for islets was RPMI 1640 containing 10% FBS, 2 mм L-glutamine, 10 mм HEPES, 100 U/ml penicillin, 100 µg/ml streptomycin, 1 mм sodium pyruvate, and 0.25 µg/ml amphotericin B.

### Ethics Statement

All animal procedures were approved by the University of California, San Diego Institutional Animal Care and Use Committee and were performed in full accordance with IACUC guidelines.

### Immunostaining

Islets were washed 3 times with D-PBS and then incubated for 1 h in 10% buffered formalin solution. Islets were washed again 3 times with D-PBS, then transferred to a 2% melted agarose solution at 55°C and spun down immediately at 5000 rpm. Once the agarose was solidified, islets were embedded in paraffin (UCSD Moores Cancer Center histology core) and then sectioned. Intact pancreases were immersed in 10% buffered formalin for 1 h, paraffin-embedded and then sectioned.

Prior to staining, pancreas sections and islets were blocked with 1% BSA in PBS-Tween (PBST). Sections were stained using a guinea-pig anti-insulin primary antibody (Dako; 1∶500 dilution) and an anti-glucagon monoclonal antibody (Sigma; 1∶500) in 1% BSA in PBST. After primary antibody incubation for 1 h, sections were washed with PBST then incubated with 1∶5000 Alexa 488 anti-guinea-pig IgG secondary antibody and 1∶5000 Alexa 594 anti-mouse IgG secondary antibody (both from Life Technologies) in 1% BSA in PBST. Sections were washed again with PBST, incubated 5 min with 1∶5000 DAPI in 1% BSA in PBST, washed with PBST, and then mounting media and plastic coverslips were applied. Images were captured using an inverted Nikon Eclipse E800 fluorescent microscope.

For automated whole-section imaging, slides were imaged using an Aperio Scanscope FL image capture device operated by the Sanford Burnham Medical Research Institute Histopathology Core. Images were converted into TIFF format and analyzed using ImagePro software (Media Cybernectics). For quantitative analysis, eight 5 µm sections spaced 80 µm apart from each pancreas were examined.

### Brightfield and EM Imaging

For brightfield imaging, isolated islets were washed 3 times with D-PBS to remove any cellular debris then imaged using an inverted Nikon Optiphot microscope. Brightfield images were analyzed using ImagePro to determine the apparent diameter of each islet, and islet cross-sectional areas were then calculated. Determination of the fraction of islets with alpha cells in the beta-cell core region was carried out as previously described [25].

Transmission electron microscopy (TEM) imaging was performed as described previously [26]. Briefly, islets were fixed in 2% glutaraldehyde in 0.1 M sodium cacodylate buffer (pH 7.4) for 4 h. Islets were then post-fixed in 1% osmium tetroxide in 0.1 M cacodylate buffer for 1 h, stained *en bloc* in 1% uranyl acetate for 1 h and then dehydrated in ethanol. Next, samples were embedded in epoxy resin and cut into 60 nM sections stained with lead nitrate and uranyl acetate. Sections were imaged using JEOL 1200 EX II transmission electron microscope and images captured with a Gatan digital camera. Images were analyzed using ImageJ software (NIH) by operators blinded as to mouse identity.

### Insulin and Protein Content

Isolated islets were washed 3 times with D-PBS then lysed with RIPA buffer containing protease inhibitors as previously described [26]. Protein content of the lysate was determined using the Bio-Rad DC protein assay and insulin content was determined by RIA (Millipore).

To determine pancreatic insulin content, pancreases were cut in half, dried, weighed and then placed into 1.5% HCl, 70% EtOH solution overnight at −20°C for insulin extraction. Tissue was then homogenized and incubated in the same solution overnight at −20°C. The tissue was then spun at 2000 rpm for 15 min at 4°C, and the supernatant then neutralized with 1 M Tris pH 7.5. Insulin content was determined by RIA.

### Analysis of Insulin Secretion

Studies of insulin secretion in static culture were performed as previously described [26]. Briefly, pooled islets from three 12–13 wk old mice were washed twice with 2.75 mM glucose Krebs-Ringer bicarbonate (KRB) buffer and allowed to equilibrate for 45 min. Islets were then treated for 1 h with KRB buffer containing either 2.75 mM glucose or 16.75 mM glucose. Media was then collected and spun down at 2000 rpm to remove debris and insulin content determined by RIA (Millipore). Islet perifusion studies were performed by the University of Washington Diabetes and Endocrinology Research Center Islet Cell and Functional Analysis Core as previously described [27]. Batches of 100 knockout and wild-type islets were loaded into parallel perifusion chambers and assessed simultaneously. The insulin content of the perifusate fractions and of representative samples of the perifused islets was measured by enzyme-linked immunosorbent assay (Alpco). Insulin secretory rate (ISR) was calculated as the fraction of islet cellular insulin content released per minute. Insulin secretion during stated time intervals was assessed as integrated area under the curve (of ISR) during the time interval. Average areas under the curve were analyzed by 2-tailed Student’s t-test.

### shRNA Delivery

Following a 24 h recovery period, isolated rat islets were infected with adenovirus vectors expressing shRNAs targeted against neuroligin-2 or, as a control, against a protein, Cav3, not expressed in rat islets (caveolin-3; absence of Cav3 message in rat islet RNA was confirmed by RT-PCR and RT-qPCR, not shown), or they were left uninfected. The viral vector, its construction from the adenovirus shuttle plasmid pDelatE1Z, and the methods by which the adenoviral vectors were purified and titers measured have been described [28]. Islets were infected using 1.9×10?7 viral particles per islet. Following a 24 h infection period, the islets were cultured for 6 more days with daily media changes. The inserted sequence targeting neuroligin-2 was: GGAGCAAGTTCAACAGCAA TTCAAGAGATTGCTGTTGAACTTGCTCC (the neuroligin-2 shRNA target sequence is underlined) and the control (Cav3 shRNA) insert sequence was: GACATTCACTGCAAGGAGATACTCGAGTATCTCCTTGCAGTGAATGTC.

### Real Time quantitative PCR

Total RNA was isolated and reverse transcribed using the Genelute RNA prep kit (Sigma). Gene-specific primers (Table 1) were designed and then used to perform qPCR using SYBR Green PCR Master mix (Applied Biosystems) and an ABI 7500 Fast Real-Time PCR Machine (Applied Biosystems). RNA samples from three wild-type and three knockout mice were analyzed in duplicate along with no-reverse transcriptase (no-RT) and water controls. Normalization of qPCR values was with values from parallel qPCR of 18S RNA. Primers were designed using Primer 3 software. Primer sequences are given in Table 1.

**Table 1:** Primers used for RT-qPCR.

Transcript	Forward primer	Reverse primer
GAD67	TGCATTTGTGAGCCAAAGAG	AATGCACAGTGTGGGTTTCA
Granuphilin	CGAGATGGAAAGGGATTTGA	AAGAGCCATTGCTTTCCAGA
Munc18	GTGAAGAAGAAGGGCGAGTG	AAATAGGGCATCTGGACACG
Neuroligin-1	GGGGATGAGGTTCCCTATGT	CCGTGGATGGCACTTTAGTT
Neurexin-1α	CGGTGTGGTGGCTTTTAAGT	CAAGCCACCCAGGTACAACT
Neurexin-2β	GTCTCGTCCAGCCTCAGC	GCCACCACTTCGAGTAAAGC
Rat neuroligin-2	CACAGAGCTCTTCACCACCA	CGATACCAGCTCCTCCTCAG

### Statistical Analysis

Data are presented as means ± SEM. Significance was analyzed by 2-tailed Student’s t-test. *P*<0.05 was considered significant.

## Results

### Analysis of Pancreas Sections

**Figure 1:**
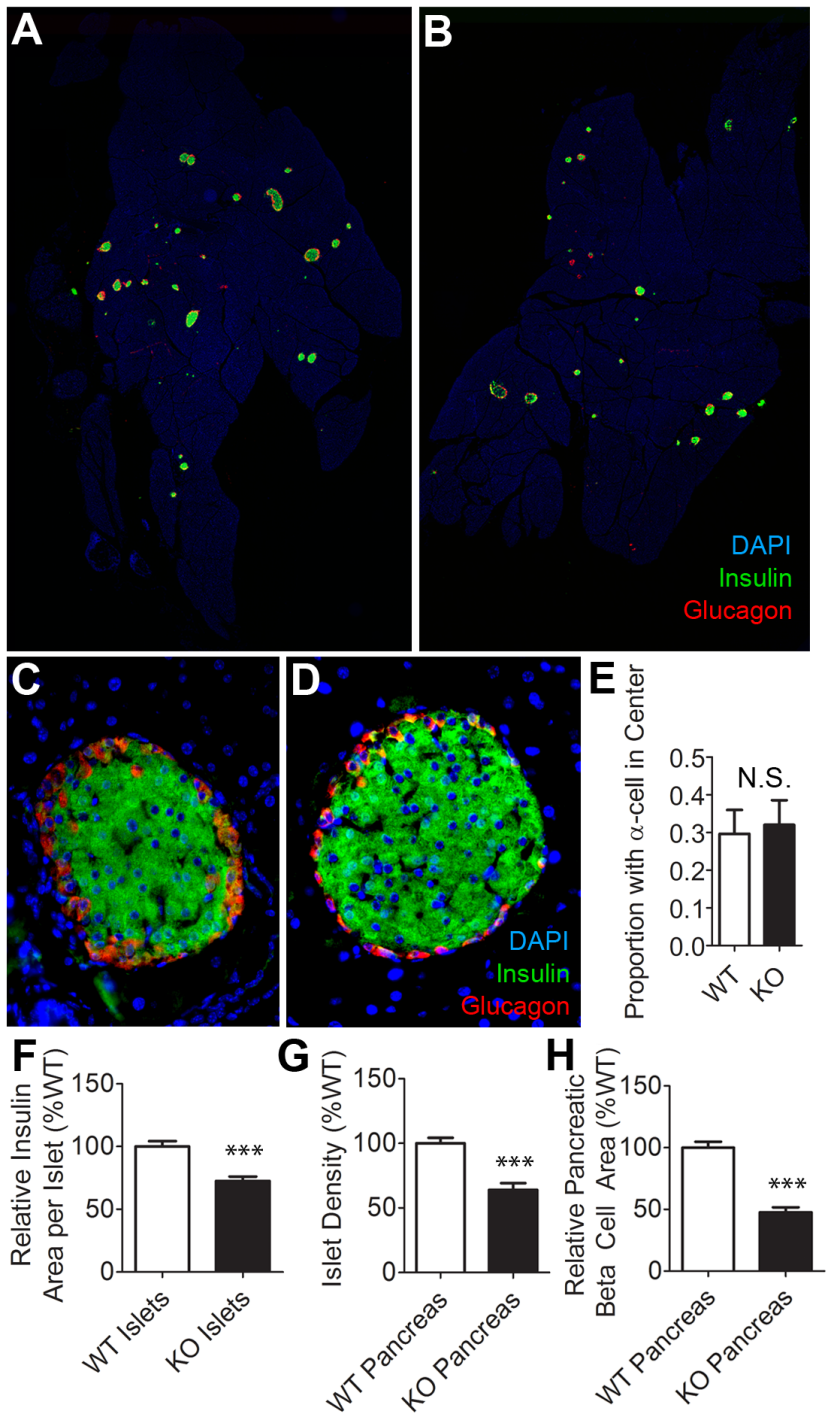
Whole section imaging and analysis of mutant and wild-type mouse pancreases. Pancreas sections from neuroligin-2 (NL-2) knockout (KO) and wild-type (WT) mice were stained for insulin (green), glucagon (red) and, as a nuclear counterstain, with DAPI (blue). *A,B* Representative whole-section images of stained pancreases: WT (A) and KO (B). *C,D*, Close-up of wild-type (C) and NL-2 KO (D) islets. *E*, Proportion of wild-type and neuroligin-2 knockout islets with alpha cells that appear to be in the beta-cell core region. *F*, Average beta-cell area per islet, shown relative to wild-type value. *G*, Islet density was measured by dividing the number of islets in each section by the total pancreas area in the section. Results are shown relative to wild-type values *H*, Total insulin area as a percentage of total pancreas section area, shown as a percentage of the wild-type value. All data are represented as mean +/− SEM (n = 8 sections at 80 µm intervals from each of 3 KO and 3 paired WT pancreases taken from 3–4 month old female littermates). ***represents a p<0.001.

We prepared pancreas sections for imaging by immunofluorescence staining using antibodies against insulin and glucagon. We then imaged the stained sections using an automated whole-section capture device. Representative sections are shown in Fig. 1. Qualitatively, wild-type (Fig. 1A) and knockout (Fig. 1B) pancreas sections could not be readily differentiated: knockout mouse islet shape and distribution appeared similar to that of wild-type mice.

The adhesion molecule N-CAM is, like neuroligin-2, expressed on the surface of both beta cells and neurons and, like neuroligin-2, is important for aspects of neuronal differentiation. N-CAM plays a key role in establishing normal islet architecture and specifically in mediating the distribution of alpha and beta cells, with alpha cells in the outer part (the mantle) and beta cells clustered in the islet center, the core [29]. In contrast to the abnormal arrangement of alpha-/beta-cells observed in N-CAM-deficient mouse islets [30], alpha- and beta-cells in neuroligin-2-deficient mice retained their characteristic mantle/core distribution (Fig. 1 A–D). The same proportion of wild-type and mutant mouse islets had alpha cells present in the beta cell core region (Fig. 1E). That approximately 30% of islets in mouse pancreas sections contain alpha cells in the core region has been previously reported [25].

During islet isolation experiments (described below), we noted that knockout islets consistently appeared smaller on average than islets from wild-type mice. The average size of islets from the mutant mice (cross-sectional islet area calculated from measured islet diameter) was 68% ±4% of that of isolated wild-type islets (data not shown). Analysis of islet beta cell area in the immunostained pancreatic sections yielded similar results (Fig. 1F). The 20% decrease in average islet insulin area observed would be indicative of a 29% reduction in volume in a hypothetical perfectly spherical islet. Decreased islet size contributed to a ∼45% decrease in the relative insulin area (area occupied by beta cells) in pancreases from the knockout mice (Fig. 1H).

Neuroligin-2 gene knockout also reduced islet number as measured by islet density, the number of discrete islets per unit of pancreatic area (Fig. 1G). This decrease in islet density also contributed to the reduced pancreatic beta cell area in the mutant mice (Fig. 1H).

### Islet Size and Insulin Content

**Figure 2:**
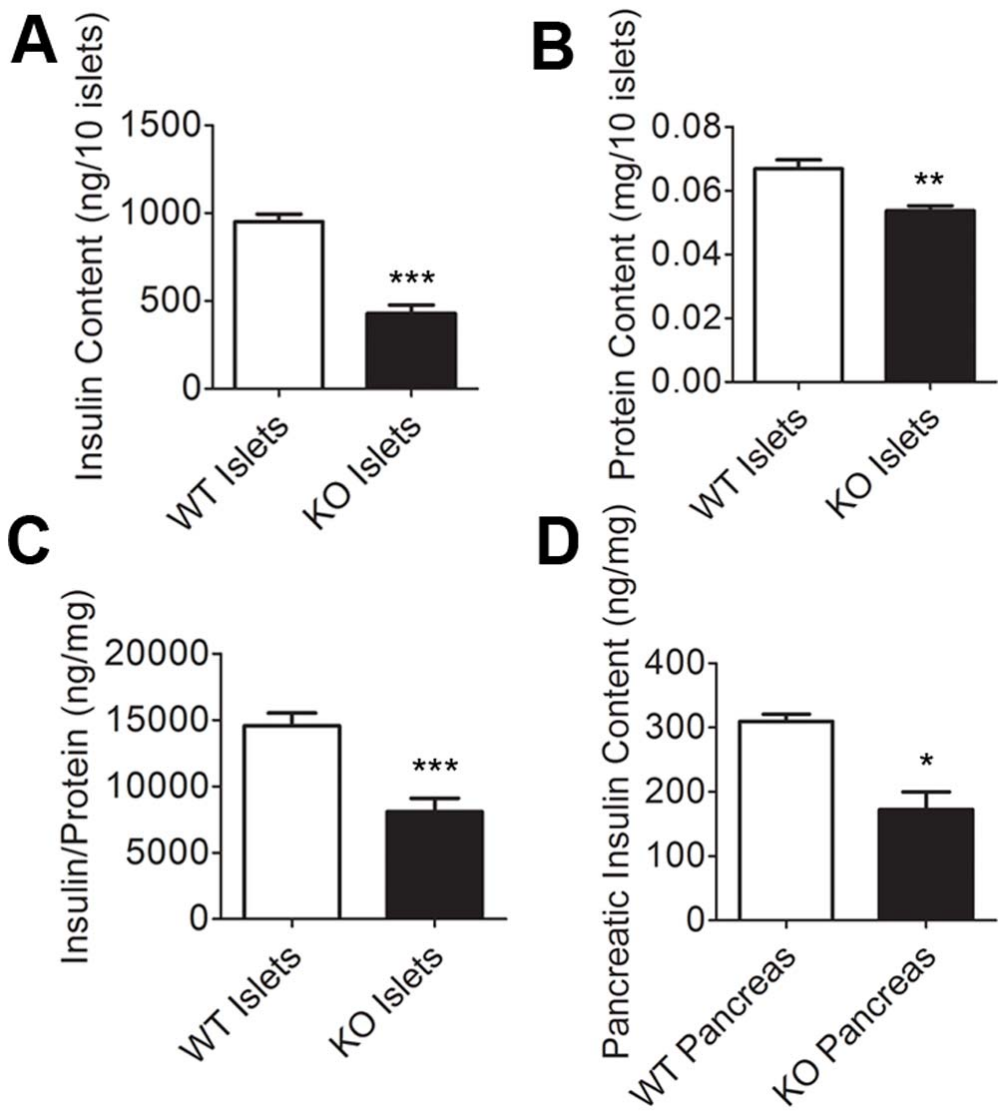
Islet and total pancreatic insulin content. Islets were isolated from NL-2 knockout (KO) and wild-type (WT) mice (white bars, WT; black bars, KO). *A*, Similarly-sized islets were hand-picked, lysed, and then insulin content was measured by RIA. *B*, Average protein content of picked islets. *C,* Islet insulin content normalized to total islet protein content. *D*, Insulin content in intact pancreases. Insulin content (ng) is shown normalized to weight (in mg) of pancreatic tissue analyzed. All data is represented as mean +/− SEM (n = 3 littermate pairs of KO and WT mice). ***represents a p<0.001; *represents p<0.05.

Our prior work suggests that transcellular neuroligin-2 interactions increase beta-cell insulin content [5]. We thus next analyzed insulin content in islets from the knockout mice. Insulin content from similarly-sized islets (Fig. 2A) was reduced in the mutant mice as was insulin content relative to total islet protein content (Fig. 2B,C). As would be predicted from the decrease in both beta-cell area (Fig. 1H) and beta-cell insulin content, pancreatic insulin content in the mutant mice was markedly lower (Fig. 2D).

### Insulin Secretion

Since our previous data indicate that extracellular interactions involving the neuroligin-2 extracellular domain help mediate the increased insulin secretion that accompanies beta-cell-beta-cell contact, we hypothesized that neuroligin-2 deficiency would impair insulin secretion in neuroligin-2 knockout islets [5]. Because the whole-body neuroligin-2 gene knockout affects brain function, weight and behavior, all of which affect glucose homeostasis in live animals, studies of insulin secretion were performed using isolated islets [31], [32]. (Conditional, beta-cell specific neuroligin-2 knockout mice that would be necessary for in vivo studies are not available nor, to the best of our knowledge, are mice with LoxP-flanked neuroligin-2 sequences).

**Figure 3:**
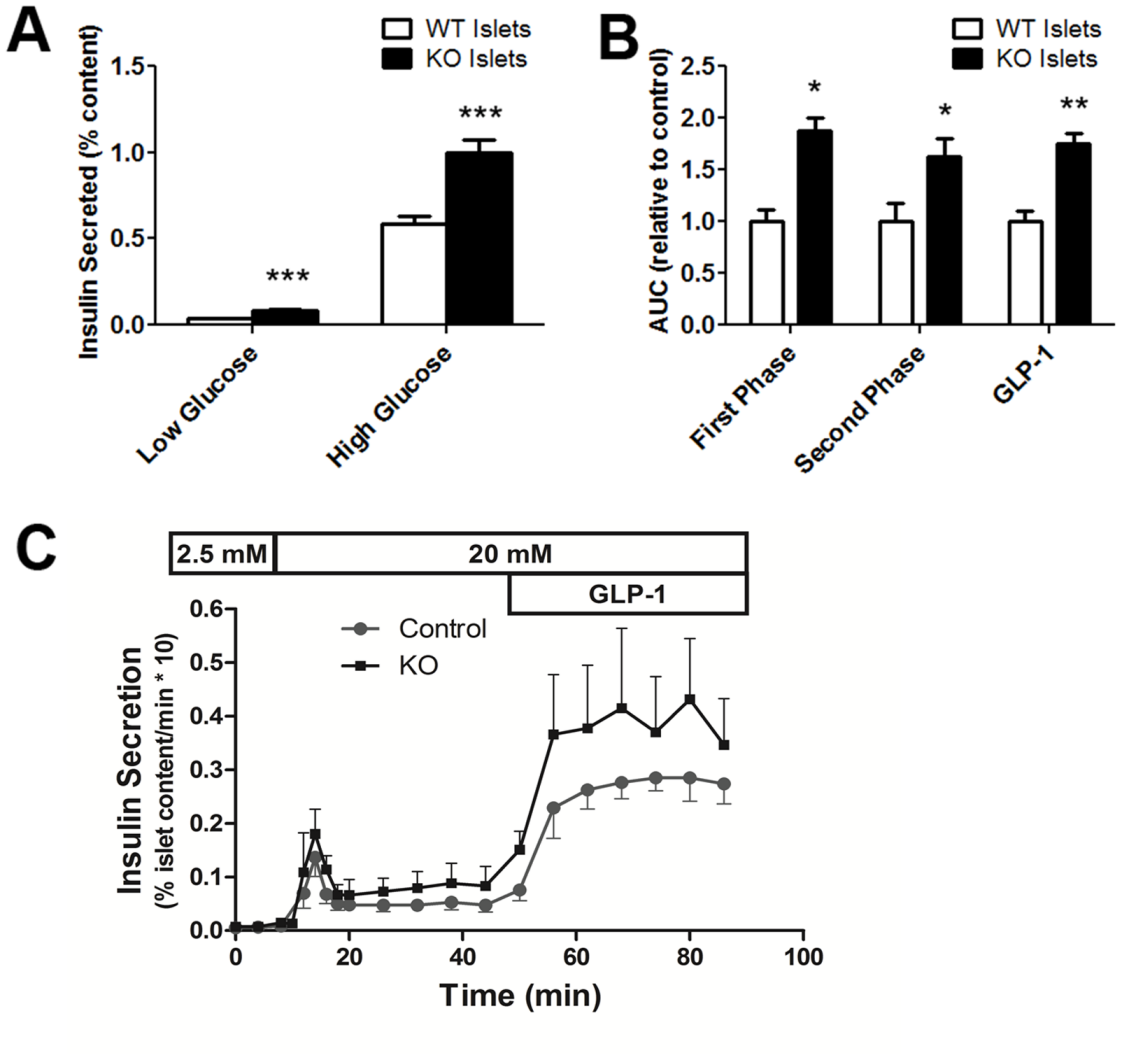
Insulin secretion by islets from mutant and wild-type mice. Islets were isolated from neurolgin-2 knockout (KO) and wild-type (WT) mice and allowed to recover overnight in culture. *A*, insulin secretion by islets in static culture. Islets from KO (black columns) and WT (white columns) mice were incubated for 1 h with either 2.75 mM (low) or 16.7 mM (high) glucose. Insulin secreted as a percentage of total islet insulin content is shown. Results are representative of three independent experiments. *B,C,* Insulin secretion by perifused islets. Islets were first perifused in buffer with 2.5 mM glucose for 30 min, with samples being collected during the last 10 min for analysis. Glucose was raised to 20 mM and perifusion was continued for another 40 min. Next, during the last 40 min, islets were perifused with 20 mM glucose and GLP-1 (100 nM). Insulin secretion was assessed as mean integrated area under the curve (AUC) of insulin secretory rate (ISR). In *B*, the relative average areas under the curve of the insulin secretory rate for first-phase insulin secretion (2 min through 8 min after stimulation with 20 mM glucose), for second-phase insulin secretion (from end of first phase up until addition of GLP-1) and for GLP-1-stimulated insulin secretion (first time point after addition of GLP-1 through end of perifusion) are shown. AUCs from KO islets (black columns) are shown relative to average AUCs from perifusions with WT islets (white columns). In *C*, the average ISR for each time point beginning with the time point 10 min prior to stimulation with 20 mM glucose is depicted. The glucose concentrations in the perifusion medium are shown above the graph, as is the time interval during which GLP-1 was also added. The ISR is the percentage of islet insulin content released per minute; the y axis shows ISR multiplied by 10 (n = 5 experiments, each with islets from pairs of KO and WT mice perifused in parallel). ***p<0.001; **, p<0.01; *p<0.05.

Contrary to what we expected, insulin secretion was enhanced in islets from the mutant mice (Fig. 3A). The increase in the fraction of cellular insulin content released was seen at both basal conditions (low glucose) and with glucose-stimulated insulin secretion (high glucose; Fig. 3A). Insulin secretion is proportional to islet insulin content: identically-sized islets from the same mouse will display different levels of glucose-stimulated insulin secretion depending on insulin content, though the percentage of insulin content released will be the same [33], [34]. Normalization to insulin content is therefore necessary for secretory efficiency to be compared when islet insulin content varies. As is common in studies of insulin secretion (e.g., see refs [35], [36], [37], [38]), insulin secretion is assessed here as a percentage of cellular insulin content (Fig. 3).

To determine if first or second phase secretion was preferentially affected, we carried out perifusion studies with islets from wild-type and mutant mice. Insulin secretory rates were monitored before and after increasing glucose in the medium from 2.5 mM to 20 mM. Insulin secretion during each phase was assessed as the mean integrated area under the curve (Fig. 3B) yielded by the separate perifusions (the mean insulin secretory rate for each time point is shown in Fig. 3C). As seen in Fig. 3B, islets lacking neuroligin-2 exhibited increased insulin secretion during both first- and second-phase insulin secretion. Increased insulin secretion by the glucose-stimulated knockout islets relative to the wild-type controls was also evident after addition of GLP-1, which activates signaling pathways that amplify glucose-stimulated insulin secretion, to the perifusion medium (Fig. 3B) [39].

**Figure 4:**
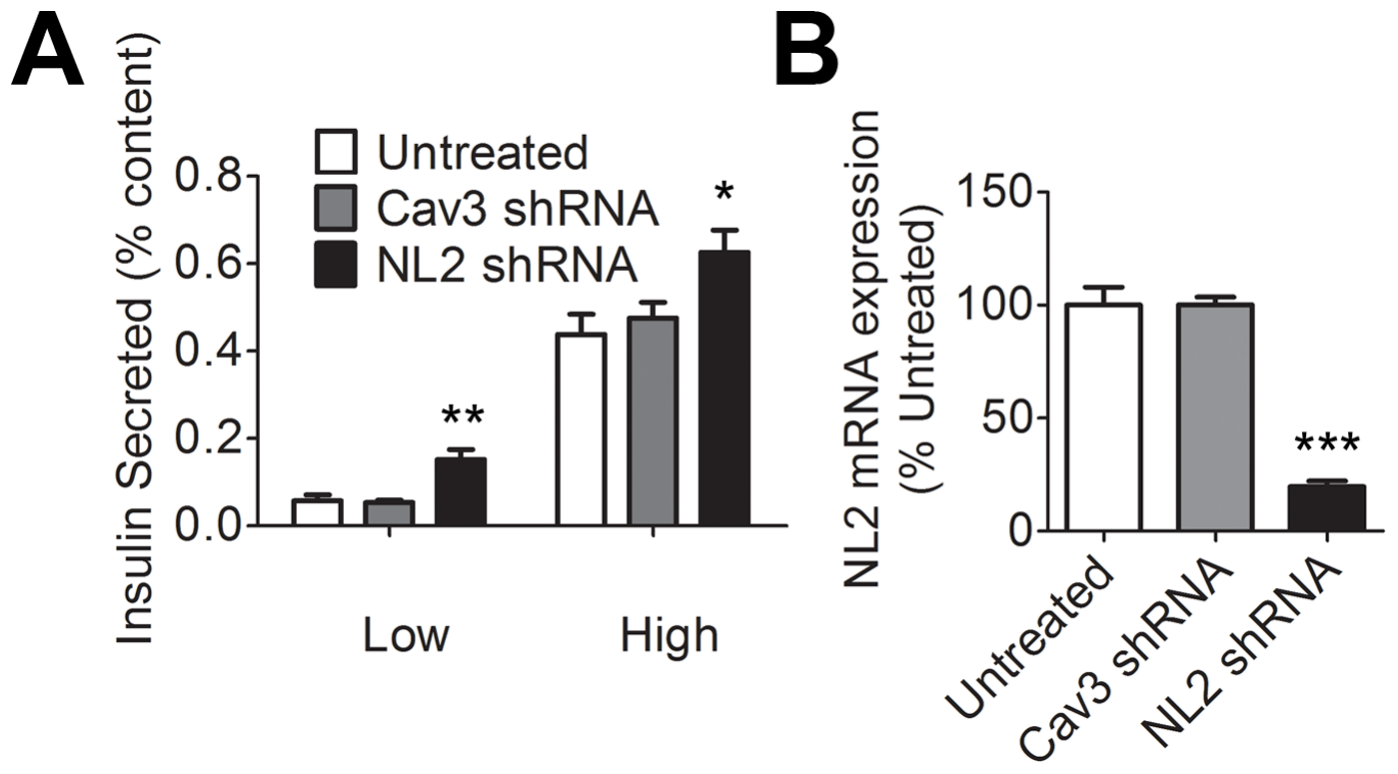
Insulin secretion after knockdown of neuroligin-2 in cultured islets. Rat islets were infected with adenoviruses expressing either shRNA against neuroligin-2 (NL2) or a control shRNA construct (Cav3) or were left uninfected (untreated). Adenovirus-containing media was removed after 24 h, and islets were cultured an additional 6 days prior to assessment of insulin secretion. *A*, Insulin secretion by islets that were either untreated or infected with adenovirus expressing shRNA was assessed after 1 h incubation in either 2.75 mM (low) or 16.7 mM (high) glucose. Insulin secreted as a percentage of total islet insulin content is shown. Results are representative of three independent experiments. *B*, NL2 message levels were assessed by real-time qPCR at the end of the culture period. Transcript levels are shown relative to the level in untreated islets. *p<0.05; **p<0.01; ***p<0.001.

To further test the link between decreased neuroligin-2 expression and increased insulin secretion, we next analyzed the effect on insulin secretion of knocking down neuroligin-2 expression in isolated islets. Rat islets were used to enable higher islet yields during isolations and also to test whether the effect on insulin secretion we observed was mouse-islet-specific. Insulin release was assessed 7 days after addition of shRNA-expressing adenovirus vectors to the islet culture medium. As seen in Fig. 4A, shRNA-mediated knockdown of neuroligin-2 mRNA increased insulin secretion. Quantitative RT-PCR was employed to assess neuroligin-2 message levels in shRNA-treated islets (Fig. 4B).

### Changes in Neuroligin-1 and Neurexin Gene Expression in Islets from Knockout Mice

**Figure 5:**
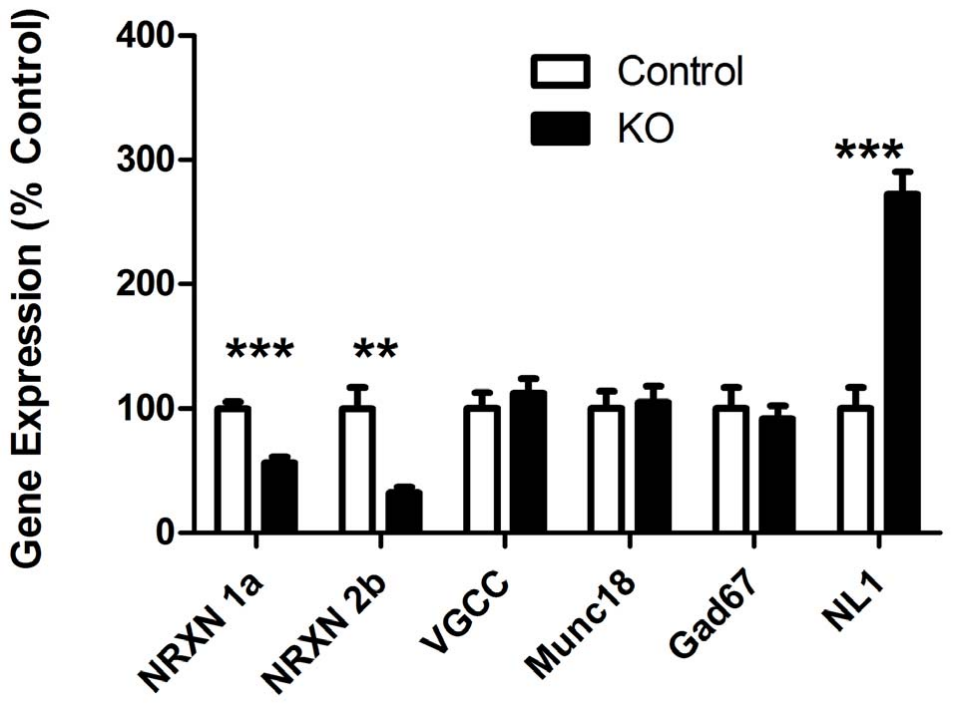
Decreased neurexin and increased neuroligin-1 transcript levels in KO islets. Real-time qPCR was used to analyze expression of neurexin-1α (NRXN 1a) and neurexin-2β (NRXN 2b); also of two representative constituents of the insulin exocytic machinery, the voltage-gated calcium channel CaV1.3 (VGCC) and Munc18; and also of the inhibitory synaptic protein GAD67; and also the expression of neuroligin-1 (NL1). Transcript levels were assessed in islets from knockout (KO; black columns) and wild-type (WT; white columns) mice. Transcript levels are shown relative to levels in the WT mice (n = 3 mice of each genotype). ***p<0.001; **p<0.01.

Neuroligin-2 is the predominant neuroligin isoform in islets and insulinoma cells [13]. Neuroligin-1 transcripts are also present in islet tissue [13]. Like neuroligin-2, neuroligin-1 drives assembly of the presynaptic secretory machinery in central nervous system synapses, though neuroligin-1 differs from neuroligin-2 in not being specific to inhibitory synapses [40]. Examination of message levels by RT-qPCR revealed a nearly 3-fold increase in the levels of neuroligin-1 transcript in islets from the mutant mice relative to wild-type islets (Fig. 5). Due to the inadequate sensitivity of available neuroligin-1 antibodies, we were unable to determine the magnitude of the presumed resultant increase in cellular neuroligin-1 protein content.

We previously found that decreased expression of neurexin, a key neuroligin binding partner, results in increased insulin secretion [26]. Since neuroligin-2-deficient islets exhibit enhanced insulin secretion, we next asked whether there was decreased expression of the two neurexin isoforms predominant in beta-cells, neurexin-1α and neurexin-2β [26]. We found that there is a substantial reduction in transcript levels of these neurexin isoforms in the mutant mice, approximately 2-fold for neurexin-1α and 3-fold for neurexin-2β (examination of endogenous neurexin protein levels was not feasible with available antibodies) (Fig. 5). In parallel, messenger RNA levels of two other, non-neurexin, representative constituents of the beta cell machinery for regulated secretion were examined: the SNARE complex-associated protein Munc18 and the voltage gated calcium channel (VGCC) CaV1.3. Transcript levels for these proteins did not change, showing that the effect on neurexin mRNA levels did not reflect a more generalized phenomenon affecting all exocytic gene expression.

Neuroligin-2 specifically drives the formation of inhibitory synapses [8], [41]. Beta cells have an inhibitory synaptic phenotype in the sense that they synthesize and package into secretory vesicles the major inhibitory neurotransmitter GABA and contain proteins that mediate GABAergic signaling and are specific markers of inhibitory synapses [42], [43], [44], [45]. The absence of neuroligin-2, however, did not alter islet transcript levels of GAD67, the larger isoform of the enzyme that catalyzes the synthesis of GABA and the predominant isoform in mouse islets (Fig. 5) [46].

### Analysis of Insulin Granule Docking

**Figure 6:**
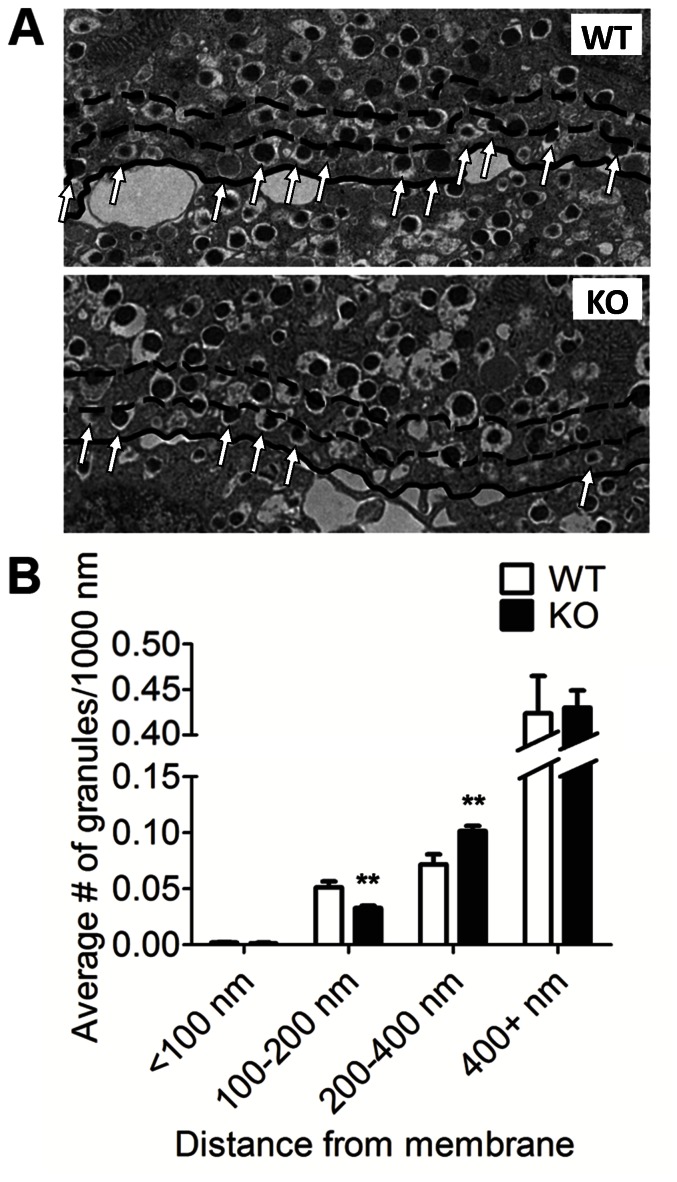
Analysis of insulin granule docking in mutant and wild-type beta cells. After a 24 h recovery period in culture, islets isolated from NL-2 KO and WT mice were fixed, pelleted and sectioned for EM. *A,* Representative electron micrographs of WT(top) and NL-2 KO (bottom) beta cells. Cell border is traced in solid black. Dashed lines are drawn 200 nM and 400 nM from the plasma membrane. Docked granules, those with centers located from 100 nm to 200 nm from the plasma membrane, are indicated by arrows. *B*, Distance from the center of each granule to the plasma membrane was measured and separated into four groups. The numbers in each group were then normalized to the total length of plasma membrane of the cell in question to determine linear density (granules per 1000 nm). Investigators were blinded to mouse identity during EM image analysis. All data are represented as mean +/− SEM from 20 cells. **, p<0.01.

Though granule docking has in the past been considered a necessary step for insulin exocytosis, more recently it has been found that docking constrains rather than facilitates insulin secretion and that increased insulin secretion accompanies reductions in insulin granule docking [26], [47], [48]. Given the decrease in neurexin expression and the unexpected enhancement of insulin secretion we observed, we next asked whether insulin granule docking was altered in the mutant mice. To assess docking, we used TEM to perform morphometric analysis on islet sections from mutant and wild type mice (Fig. 6). We measured the linear density of granules docked along the plasma membrane in the manner described in previous studies of insulin granule docking [49], [50]. Docked granules were quantified, as has been described previously, by counting granules with centers 100–200 nm from the plasma membrane [47], [48]. Complementing our preliminary analysis indicating that the overall fraction of docked insulin granules is lower in the mutant mice (6% in mutant vs. 9% in wild-type, p<0.001) [5], we found that the number of docked granules per unit length of plasma membrane was less in the neuroligin-2-deficient mice than in the wild-type controls (Fig. 6B). In contrast, the linear density of granules close to the membrane, with centers 200–400 nm away, but not docked was greater in the mutant mice than in the controls.

## Discussion

The effects of neuroligin-2 knockout on brain and central synapse formation, on behavior, on sociability and on other parameters related to brain function have previously been described [11], [14], [15], [16], [17]. In addition to its presence in inhibitory synapses in the central nervous system, neuroligin-2 is also expressed by pancreatic beta cells [13]. We recently found that the extracellular domain of neuroligin-2 engages in transcellular interactions that promote insulin secretion. These studies were carried out in a co-culture system analogous to the co-culture model in which the synaptogenic function of the neuroligins was first discovered [8], [51]. Culturing pancreatic beta cells in contact with neuroligin-2 expressing HEK293 cells enhanced basal and stimulated insulin secretion, and this was at least in part due to an effect of neuroligin-2 on maturation of the beta-cell secretory machinery [5]. We also found that neuroligin-2 interactions increased INS-1 cell proliferation in the co-culture system [5]. The proliferative properties of INS-1 cells are very different from those of primary beta cells, so while this finding was intriguing, we drew no conclusions regarding a possible effect of neuroligin-2 on beta cell proliferation. Nevertheless, this result made us wonder whether there would be evidence of reduced beta-cell formation in neuroligin-2 knockout mice.

Our work here was guided by two objectives. Given the importance of transcellular interactions involving neuroligin-2 to insulin secretion in the co-culture system, our first objective was to test whether loss of neuroligin-2 causes impaired insulin secretion in islets from neuroligin-2-deficient mice. The second objective was to test whether neuroligin-2 influences islet formation, thereby adding to our knowledge of the phenotypic consequences of neuroligin-2 knockout and following-up on our observation that neuroligin-2 increases INS-1 β cell proliferation in co-culture experiments [5].

Loss of neuroligin-2 expression had significant effects on islet formation in the mutant mice. The islets in the neuroligin-2-deficient mice were, on average, smaller in size and reduced in number: fewer islets formed on average in any given pancreatic cross-sectional area. The neuronal cell surface may display proteins that enhance proliferation of cultured beta-cells, and it will be of interest in future work to determine if neuroligin-2 is one of these proteins [52]. Our prior work suggested that transcellular-neuroligin-2 interactions increase beta cell insulin content [5]. Consistent with this, beta cell insulin content was lower in the mutant mice.

The results reported here are in accord with our earlier results in that neuroligin-2 was found to influence beta-cell function. However, in contrast to our studies in the co-culture model in which we found that neuroligin-2 promoted insulin secretion, in islets from the knockout mice, increased insulin secretion was observed in the absence of neuroligin-2. This was true under both basal and stimulated conditions. Neuroligin-2 knockdown in isolated rat islets yielded a parallel result: increased insulin secretion at both low and stimulating glucose concentrations. The differences between these results and our prior results are most likely due to the significant differences between the model systems we employed [5]. In the co-culture system, the effects of neuroligin-2 interactions over 24 h were investigated. In contrast, in the mutant mice, neuroligin-2 was absent continuously, throughout development, and insulin secretion by isolated rat islets was analyzed 7 days after the anti-neuroligin-2 shRNA-expressing adenovirus was added to the culture medium. Additionally, in the co-culture system, only the transcellular effects of the neuroligin-2 extracellular domain (which was displayed on the surface of HEK293 cells) were investigated. In the mutant mice and shRNA-treated rat islets, the loss of the entire neuroligin-2 protein impacted both interactions involving the extracellular domain as well as any cytoplasmic interactions involving the protein’s intracellular domain.

The neurexins are major neuroligin binding partners [8]. The binding of presynaptic neurexins by postsynaptic neuroligins across the synaptic cleft is essential for normal neurotransmitter secretion [7], [8]. Neurexins are also necessary for normal secretory function in beta cells [26]. Neurexin-1α helps mediate insulin granule docking, a step in the exocytic pathway where the granules can be trapped just prior to membrane fusion and insulin release [26], [47], [48]. Decreased neurexin-1α expression frees insulin granule exocytosis from this constraining step and thus increases insulin secretion [26]. Decreased neurexin-2β expression similarly increases insulin secretion, probably in the same manner [26]. Here we found that neurexin-1α and -2β transcript levels are markedly reduced in islets from the mutant mice. The increased insulin secretion resulting from loss of neuroligin-2 expression, then, is probably caused at least in part by decreased neurexin expression. Other factors may also have contributed to the observed changes in insulin secretion, including the loss from the beta cell cytoplasm of the neuroligin-2 intracellular domain. The reduction in insulin granule docking observed in beta cells from the mutant mice, however, is evidence for a causal role of decreased neurexin expression.

The effects we observed on neurexin and neuroligin-1 mRNA levels have not previously been reported in studies using neuroligin-2 knockout mice, and the means by which neuroligin-2 levels influence the transcription or transcript stability of neurexin and neuroligin-1 remain to be determined. Together, our results suggest that compensatory mechanisms enable the beta cells to counter neuroligin-2 deficiency by means including increased expression of neuroligin-1, which may function as a substitute for neuroligin-2, and enhancement of insulin secretory efficiency brought about by reduced neurexin expression. These mechanisms may be triggered directly by the loss of neuroligin-2 expression and/or by the associated reductions in insulin secretory capacity, beta cell number or beta cell insulin content.

Since neuroligin-2 is a trigger for nascent synapses to differentiate into inhibitory rather than excitatory synapses, we hypothesized that GAD67 would decrease in the absence of neuroligin-2 [7], [11]. Loss of neuroligin-2 expression, however, did not impact beta-cell GAD67 transcript levels. This suggests that neuroligin-2 does not drive the acquisition by the beta-cell of at least some of its characteristic GABAergic synapse-like phenotypic features [43], [44], [45].

In summary, our results show that neuroligin-2 expression is necessary for normal islet and specifically beta-cell formation: in the absence of neuroligin-2, islets are smaller and fewer in number, with reduced beta cell insulin content. To determine with certainty whether the effects we observed of neuroligin-2 deletion on islet formation were due solely to loss of the protein in the islets, future studies using a conditional, islet-specific neuroligin-2 knockout mouse will be necessary. Our results confirm that, as previously found in our co-culture system, neuroligin-2 influences insulin secretion. The earlier co-culture studies revealed that transcellular neuroligin-2 interactions can drive assembly of the insulin secretory machinery and increase insulin secretion. Here we found that neuroligin-2 is necessary for normal levels of neurexin expression and insulin granule docking, and that, perhaps because of this, insulin secretion is increased in islets lacking neuroligin-2. Our findings provide further evidence that neuroligin-2 plays an integral role in the maintenance of normal beta cell function.
